# Supplementary material for: The N-Terminal Region of Yeast Protein Phosphatase Ppz1 Is a Determinant for Its Toxicity
Source: Int J Mol Sci. 2020 Oct 19;21(20):7733. doi: 10.3390/ijms21207733 (PMC7590015; doi:10.3390/ijms21207733)
Supplement: Supplementary file 1 [file ijms-21-07733-s001.zip › Table S1 (oligonuclotides).pdf]

Table S1. Oligonucleotides used in this work

Name	Sequence	Use
OMLM3	CGGGATCCAAAATGGGTAATTCAAGTTCAAAATC	Amplification of C-terminally HA-tagged *PPZ1* ORF
OMLM4	TGCACTGCAGTTACTGTTGAGATTCGTTATCATTTG	Amplification of untagged *PPZ1* ORF
OMLM7	GGTACCATGGGTAATTCCGG	Amplification of *PPZ2* ORF and C-terminally HA-tagged *PPZ2* ORFs
OMLM8	CTCCTGCAGCGATTGGCTAATTTACG	Amplification of *PPZ2* and N-terminally HA-tagged *PPZ2* ORFs
OMLM9	CGGGATCCAAAATGGCTAATTCAAGTTCAAAATCTTCGAAA	Amplification of untagged *PPZ1*^{G2A} *variant*
OMLM11	TGACTGCAGTTATTTTTTCTTTCTACCCCCAGC	Amplification of N-terminally HA-tagged *GLC7* ORF
HA_PPZ2_Fw_KpnI	CTCGGTACCATGTACCCATACGATGTTCCAGATTACGCTGGTAATTCC GGTTCGAAACAAC	Amplification of N-terminally HA-tagged *PPZ2* ORF
HA_GLC7_Fw_BamHI	CGGGATCCATGTACCCATACGATGTTCCAGATTACGCTGACTCACAAC CAGTTGACG	Amplification of N-terminally HA-tagged GLC7 ORF
OCCP1	TGCACTGCAGTTAAGCGTAATCTGGAACATCGTATGGATACTGTTGAG ATTCGTTATCATTTG	Amplification of C-terminally HA-tagged *PPZ1* ORF
OCCP2	TGCACTGCAGTTAAGCGTAATCTGGAACATCGTATGGATAGCGATTGG CTAATTTACG	Amplification of C-terminally HA-tagged *PPZ2* ORF
PPZ2_Cter_BspEI_Fw	AGTCCGGAGTCGAAATGAAAAGGAAAAAACC	Generation of pCM190-PPZ1:2-HA
PPZ2_Nter_BspEI_Rv	AGTCCGGACATAGTATTAGTCCTAGATCCATTATC	Generation of pCM190-PPZ2:1-HA
PPZ1_Fw_-470_KpnI	ATGGTACCAACTCAAGGAGAAGGGCA	Amplification of the *PPZ1-GFP* ORF
EGFP_mCherry_Rv_HindIII	ATAAGCTTCTGTTATCCCTAGCGGATCT	Amplification of the *PPZ1-GFP* ORF
Ppz1 G2A_Fw	CTTTCCTTCCTTTTCAAAATGGCTAATTCAAGTTCAAAATCTTCGAAA	Quickchange mutation of Gly2 to Ala from *PPZ1-GFP*
Ppz1 G2A_Rv	TTTCGAAGATTTTGAACTTGAATTAGCCATTTTGAAAAGGAAGGAAAG	Quickchange mutation of Gly2 to Ala from *PPZ1-GFP*
S3_Ppz1_Ct_Fluo_Fw	CAGCAAATGATGGAAACAAGCATCACAAATGATAACGAATCTCAACAG CGTACGCTGCAGGTCGAC	Construction of strain DVS001
S2_Ppz1_Ct_Fluo_Rev	ATGAAATAATGAATATAATACTTCTGGATTTTTTAGTCAGTAAATTTA ATCGATGAATTCGAGCTCG	Construction of strain DVS001
