# Supplementary material for: The N-Terminal Region of Yeast Protein Phosphatase Ppz1 Is a Determinant for Its Toxicity
Source: Int J Mol Sci. 2020 Oct 19;21(20):7733. doi: 10.3390/ijms21207733 (PMC7590015; doi:10.3390/ijms21207733)
Supplement: Supplementary file 1 [file ijms-21-07733-s001.zip › Figs S1-S6.pdf]

Supplementary Figure 1. Sequences of proteins described in this work. The color code is the same used in Figure 1. Catalytic domains are denoted in bold. The HA epitope tag is indicated in dark blue background.

Ppz1-HA

MGNSSSKSSKKDSHSNSSSRNPRPQVSRTETSHSVKSAKSNKSSRSRRSLPSSSTTNTNSNVPD
PSTPSKPNLEVNHQRHSSHTNRYHFPSSSHSHSNSQNELLTTPSSSSTKRPSTSRRSSYNTKAA
ADLPPSMIQMEPKSPILKTNNSSTHVSKHKSSYSSTYYENALTDDDNDDKDNDISHTKRFSRSS
NSRPSSIRSGSVSRRKSDVTHEEPNNGSYSSNNQENYLVQALTRSNSHASSLHSRKSSFGSDGN
TAYSTPLNSPGLSKLTDHSGEYFTSNSTSSLNHHSSRDIYPSKHISNDDDIENSSQLSNIHASM
ENVNDKNNNITDSKKDPNEEFNDIMQ**SSGNKNAPKKFKKPIDIDETIQKLLDAGYAAKRTKNVC**
LKNNEILQICIKAREIFLSQPSLLELSPPVKIVGDVHGQYGDLLRLFTKCGFPPSSNYLFLGDY
VDRGKQSLETILLLFCYKIKYPENFFLLRGNHECANVTRVYGFYDECKRRCNIKIWKTFIDTFN
TLPLAAIVAGKIFCVHGGLSPVLNSMDEIRHVVRPTDVPDFGLINDLLWSDPTDSPNEWEDNER
GVSYCYNKVAINKFLNKFGFDLVCRAHMVVEDGYEFFNDRSLVTVFSAPNYCGEFDNWGAVMSV
SEGLLCSFELLDPLDSAALKQVMKKGRQERKLANQQQQMMETSITNDNESQQYPYDVPDYA*

Ppz2-HA

MGNSGSKQHTKHNSKKDDHDGDRKKTLDLPPLTKSDTTHSLKSSRSLRSLRSKRSEASLASNVQ
AQTQPLSRRSSTLGNGNRNHRRSNNAPITPPNNHYLTSHPSSSRRLSSSSRRSSMGNNNNSELP
PSMIQMEPKSPILKNSTSMHSTSSFNSYENALTDDDDDRGDDGGESPSMAKVTRINTSSSADRG
SKRTPLRRHNSLQPEKGVTGFSSTSSKLRRRSDNTLPASYPLNAEAGGNGSDYFSNRSNSHASS
RKSSFGSTGNTAYSTPLHSPALRKMSSRDNDDSGDNVNGRGTSPIPNLNIDKPSPSASSASKRE
YLSAYPTLAHRDSSSSLSPRGKGQRSSSSSSSSQRIYVSPPSPTGDFVHGSCADGDNGSRTNT**M**
VEMKRKKPVRPVDIDEIIQRLLDAGYAAKRTKNVCLKNSEIIQICHKARELFLAQPALLELSPS
VKIVGDVHGQYADLLRLFTKCGFPPMANYLFLGDYVDRGKQSLETILLLLCYKIKYPENFFLLR
GNHECANVTRVYGFYDECKRRCNIKIWKTFVDTFNTLPLAAIVTGKIFCVHGGLSPVLNSMDEI
RHVSRPTDVPDFGLINDLLWSDPTDSSNEWEDNERGVSFCYNKVAINKFLNKFGFDLVCRAHMV
VEDGYEFFNDRSLVTVFSAPNYCGEFDNWGAVMTVSEGLLCSFELLDPLDSTALKQVMKKGRQE
RKLANRYPYDVPDYA*

Ppz1:2-HA

MGNSSSKSSKKDSHSNSSSRNPRPQVSRTETSHSVKSAKSNKSSRSRRSLPSSSTTNTNSNVPD
PSTPSKPNLEVNHQRHSSHTNRYHFPSSSHSHSNSQNELLTTPSSSSTKRPSTSRRSSYNTKAA
ADLPPSMIQMEPKSPILKTNNSSTHVSKHKSSYSSTYYENALTDDDNDDKDNDISHTKRFSRSS
NSRPSSIRSGSVSRRKSDVTHEEPNNGSYSSNNQENYLVQALTRSNSHASSLHSRKSSFGSDGN
TAYSTPLNSPGLSKLTDHSGEYFTSNSTSSLNHHSSRDIYPSKHISNDDDIENSSQLSNIHASM
ENVNDKNNNITDSKKDPNEEFNDIMQSSG**VEMKRKKPVRPVDIDEIIQRLLDAGYAAKRTKNVC**
LKNSEIIQICHKARELFLAQPALLELSPSVKIVGDVHGQYADLLRLFTKCGFPPMANYLFLGDY
VDRGKQSLETILLLLCYKIKYPENFFLLRGNHECANVTRVYGFYDECKRRCNIKIWKTFVDTFN
TLPLAAIVTGKIFCVHGGLSPVLNSMDEIRHVSRPTDVPDFGLINDLLWSDPTDSSNEWEDNER
GVSFCYNKVAINKFLNKFGFDLVCRAHMVVEDGYEFFNDRSLVTVFSAPNYCGEFDNWGAVMTV
SEGLLCSFELLDPLDSTALKQVMKKGRQERKLANRYPYDVPDYA*

Ppz2:1

MGNSGSKQHTKHNSKKDDHDGDRKKTLDLPPLTKSDTTHSLKSSRSLRSLRSKRSEASLASNVQ
AQTQPLSRRSSTLGNGNRNHRRSNNAPITPPNNHYLTSHPSSSRRLSSSSRRSSMGNNNNSELP
PSMIQMEPKSPILKNSTSMHSTSSFNSYENALTDDDDDRGDDGGESPSMAKVTRINTSSSADRG
SKRTPLRRHNSLQPEKGVTGFSSTSSKLRRRSDNTLPASYPLNAEAGGNGSDYFSNRSNSHASS
RKSSFGSTGNTAYSTPLHSPALRKMSSRDNDDSGDNVNGRGTSPIPNLNIDKPSPSASSASKRE
YLSAYPTLAHRDSSSSLSPRGKGQRSSSSSSSSQRIYVSPPSPTGDFVHGSCADGDNGSRTNTM
SGNKNAPKKFKKPIDIDETIQKLLDAGYAAKRTKNVCLKNNEILQICIKAREIFLSQPSLLELS
PPVKIVGDVHGQYGDLLRLFTKCGFPPSSNYLFLGDYVDRGKQSLETILLLFCYKIKYPENFFL
LRGNHECANVTRVYGFYDECKRRCNIKIWKTFIDTFNTLPLAAIVAGKIFCVHGGLSPVLNSMD
EIRHVVRPTDVPDFGLINDLLWSDPTDSPNEWEDNERGVSYCYNKVAINKFLNKFGFDLVCRAH
MVVEDGYEFFNDRSLVTVFSAPNYCGEFDNWGAVMSVSEGLLCSFELLDPLDSAALKQVMKKGR
QERKLANQQQQMMETSITNDNESQQYPYDVPDYA*

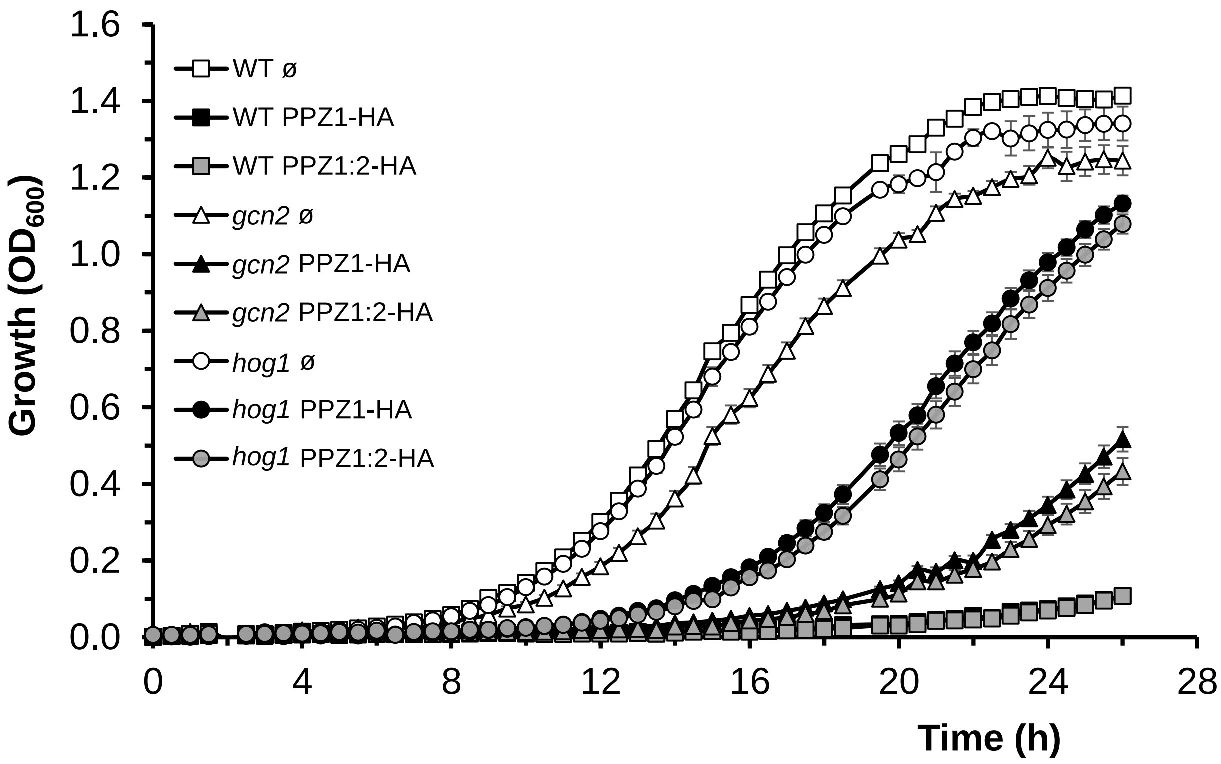

Figure S2. Growth profiles of cells overexpressing Ppz1-HA and Ppz1:2-HA and carrying the *gcn2* or the *hog1* mutations. BY4741 cells (WT) and its *gcn2* and *hog1* derivatives were transformed with the indicated pCM190-derived plasmids and grown in liquid medium as described in Material and Methods (except initial OD was 0.002). The OD_{600} was determined every 30 min. Data is presented as the mean ± SEM of three independent experiments made by triplicate.

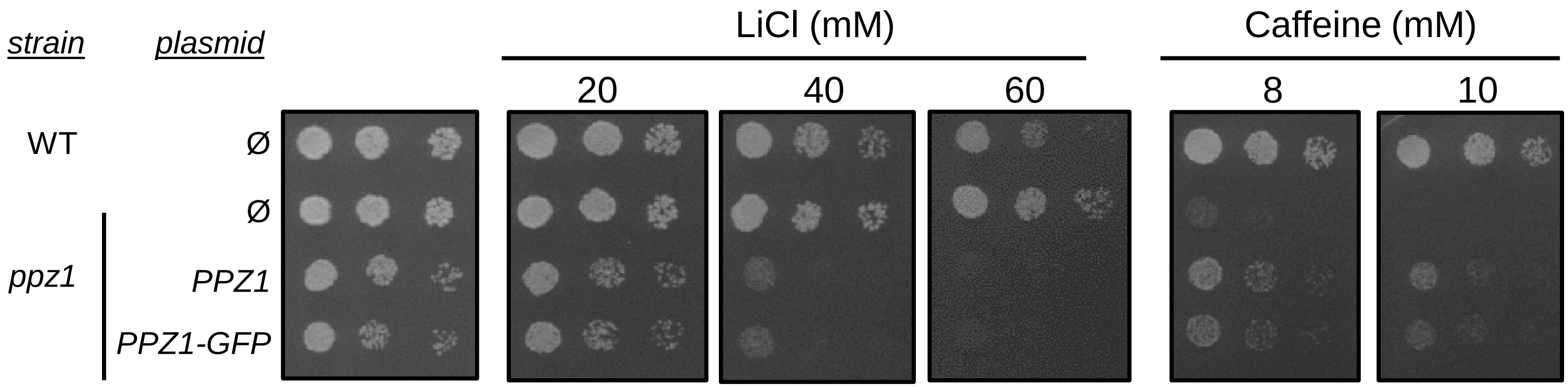

Figure S3. Functional analysis of the C-terminally GFP-tagged Ppz1 phosphatase. The indicated plasmids (YEplac195-based) were introduced in the wild type (WT) DBY746 strain and its isogenic *ppz1* derivative and tested on SC (lacking uracil) plates for tolerance to LiCl and caffeine. Plates were incubated for 3 days. Ø, empty YEplac195 plasmid.

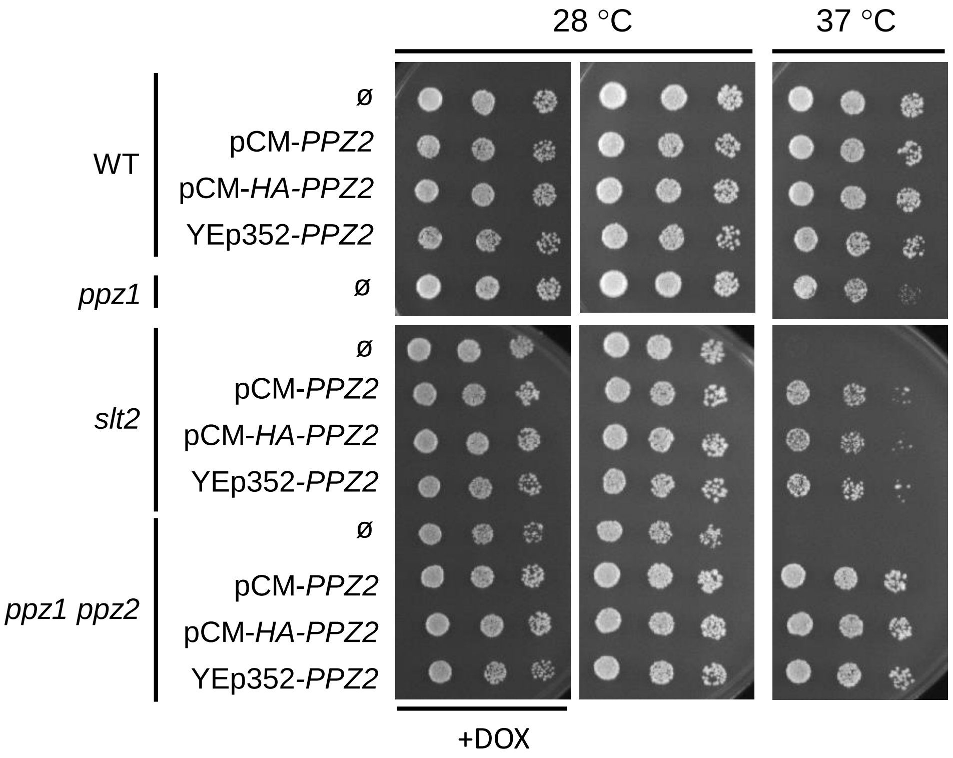

Figure S4. Functional analysis of the N-terminally HA-tagged Ppz2 phosphatase. The indicated plasmids (pCM is pCM190) were introduced in the wild type (WT) BY4741 strain and its isogenic derivatives and tested for tolerance to high temperature. Plates were incubated for 3 days. Ø, empty pCM190 plasmid .

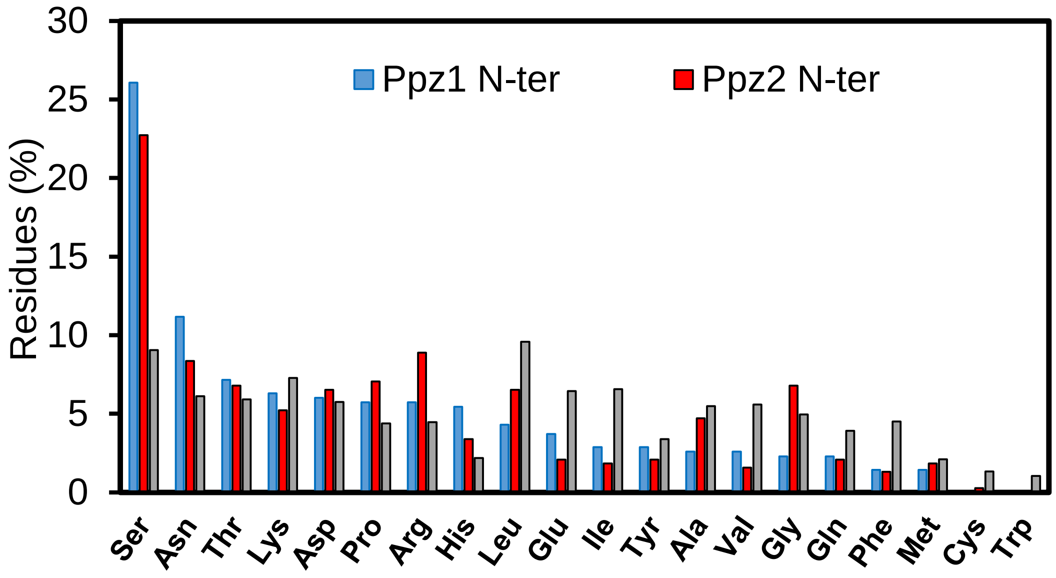

Figure S5. Percent aminoacidic composition of the N-terminal region of Ppz1 (349 residues) and Ppz2 (383 residues) in comparison with the whole yeast proteome (grey bars).

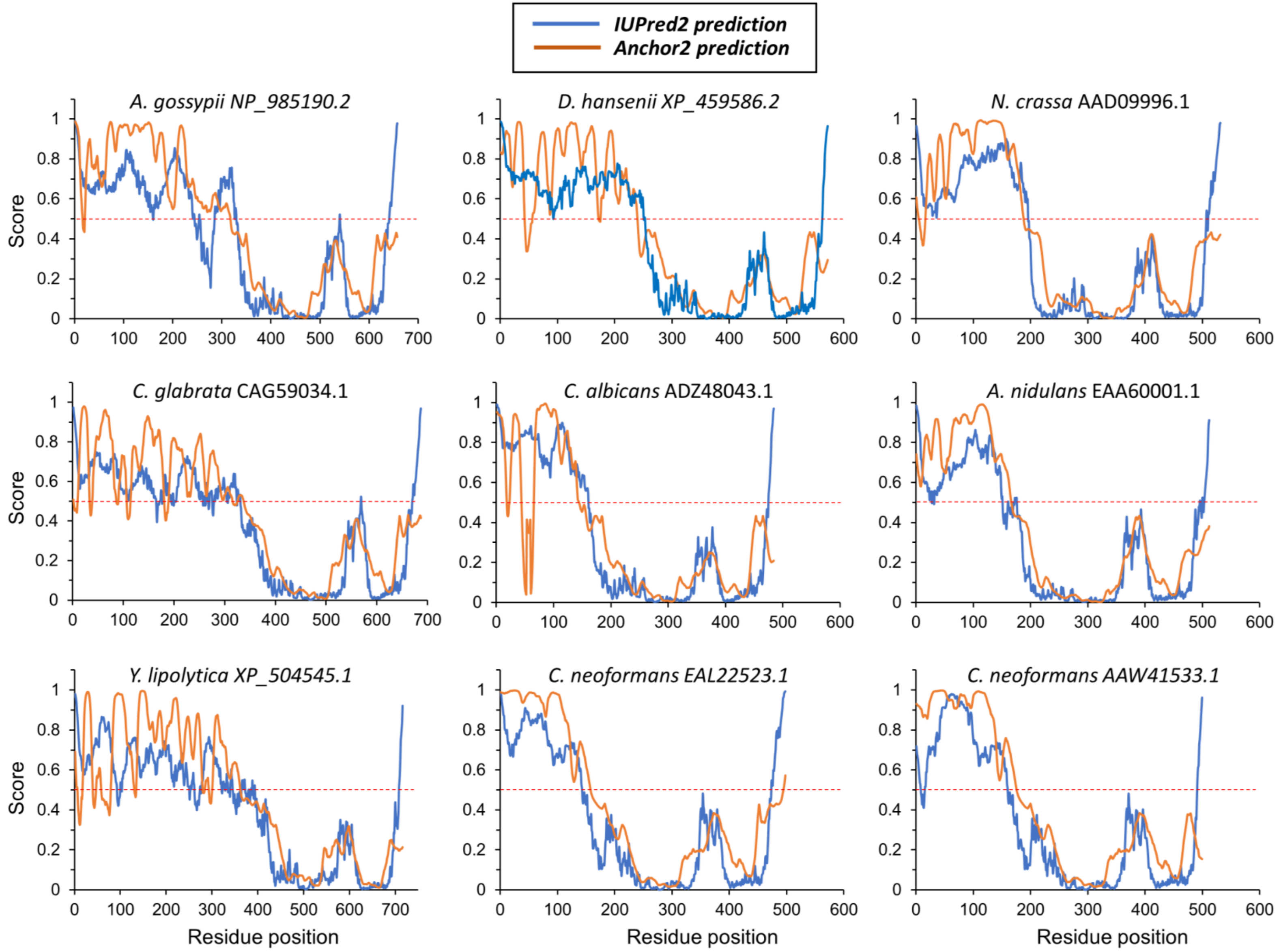

Figure S6. Prediction of Intrinsically Disordered (IDRs) and folding upon binding regions for Ppz phosphatases from diverse fungi. A) Prediction of IDRs according the IUPred2 software. B) Prediction of disordered binding regions based in the Anchor2 software. The 0.5 cut-off (discontinuous line) corresponds to 5% false positive prediction on IDRs or ordered protein segments.
